# Mycoplasmosis in Ferrets

**DOI:** 10.3201/eid1811.120072

**Published:** 2012-11

**Authors:** Matti Kiupel, Danielle R. Desjardins, Ailam Lim, Carole Bolin, Cathy A. Johnson-Delaney, James H. Resau, Michael M. Garner, Steven R. Bolin

**Affiliations:** Michigan State University, Lansing, Michigan, USA (M. Kiupel, D.R. Desjardins, A. Lim, C. Bolin, S.R. Bolin);; Eastside Avian and Exotic Animal Medical Center, Kirkland, Washington, USA (C.A. Johnson-Delaney);; Van Andel Research Institute, Grand Rapids, Michigan, USA (J.H. Resau);; and Northwest ZooPath, Monroe, Washington, USA (M.M. Garner)

**Keywords:** ferret, Mustelidae, respiratory tract disease, disease outbreak, Mycoplasma, mycoplasmosis, bacteria

## Abstract

We report an outbreak of severe respiratory disease associated with a novel *Mycoplasma* species in ferrets. During 2009–2012, a respiratory disease characterized by nonproductive coughing affected ≈8,000 ferrets, 6–8 weeks of age, which had been imported from a breeding facility in Canada. Almost 95% became ill, but almost none died. Treatments temporarily decreased all clinical signs except cough. Postmortem examinations of euthanized ferrets revealed bronchointerstitial pneumonia with prominent hyperplasia of bronchiole-associated lymphoid tissue. Immunohistochemical analysis with polyclonal antibody against *Mycoplasma bovis* demonstrated intense staining along the bronchiolar brush border. Bronchoalveolar lavage samples from 12 affected ferrets yielded fast-growing, glucose-fermenting mycoplasmas. Nucleic acid sequence analysis of PCR-derived amplicons from portions of the 16S rDNA and RNA polymerase B genes failed to identify the mycoplasmas but showed that they were most similar to *M. molare* and *M. lagogenitalium.* These findings indicate a causal association between the novel *Mycoplasma* species and the newly recognized pulmonary disease.

## Abstract 2

A newly recognized respiratory disease of domestic ferrets is associated with a novel *Mycoplasma* species.

The number of pet ferrets in the United States has grown rapidly, from an estimated 800,000 in 1996 (*1*) to an estimated 7–10 million in 2007 (*2*). Also in the United States, ferrets have become the third most common household pet; their popularity as a pet in Europe is similar (*3*). The common respiratory diseases in pet ferrets are caused by viruses; canine distemper is probably the most virulent (*4*). Ferrets also are highly susceptible to human influenza virus, but disease is rarely severe (*5*,*6*). Bacteria rarely cause disease outbreaks in ferret populations, but they do cause disease in individual ferrets (*7*–*9*).

In 2007, in the state of Washington, USA, an outbreak of respiratory disease characterized by a dry, nonproductive cough was observed in 6- to 8-week-old ferrets at a US distribution center of a commercial pet vendor (Video). Over a 4-year period, ≈8,000 ferrets, equal numbers of both sexes, were affected. Every 2–3 weeks, kits had been shipped in groups of 150–200 from a commercial breeding facility in Canada to the distribution center. At 5 weeks of age, before shipment to the distribution center, each kit received a single vaccination for distemper (DISTEM R-TC; Schering Plough, Kenilworth, NJ, USA).

**Video:**
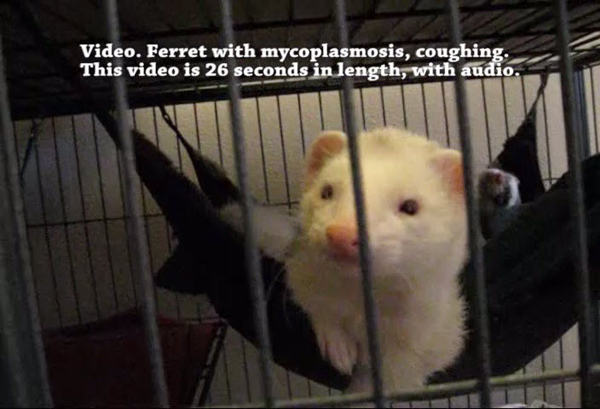
Ferret with mycoplasmosis, coughing. This video is 26 seconds in length, with audio.

Direct Video Link: http://streaming.cdc.gov/vod.php?id=cb2ddf6bcc243162bf22db00a0e82d7320120918082004000

Some ferrets exhibited hemoptysis, labored breathing, sneezing, and conjunctivitis. Almost 95% of the ferrets were affected, but almost none died. Symptomatic ferrets were selected from each shipment for testing; results of heartworm screening, PCR and serologic testing for distemper, and serologic testing for influenza virus were negative. Cytologic examination of bronchioalveolar lavage (BAL) samples yielded few inflammatory cells. Thoracic ultrasonography found no abnormalities. Thoracic radiographs showed a mild bronchointerstitial pattern with peribronchial cuffing (Figure 1). Complete blood counts and chemistry results were within reference ranges (*10*,*11*).

**Figure 1:**
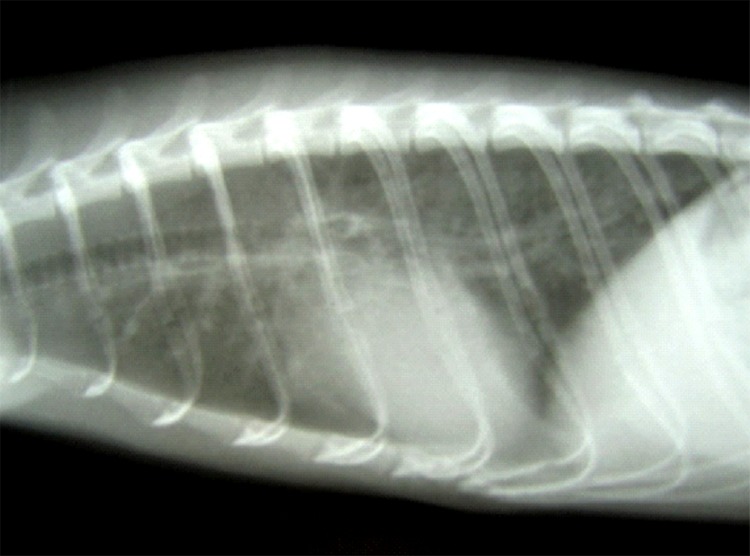
Lateral radiographic view of the thorax from a 2-year-old ferret with cough and labored breathing, showing a bronchointerstitial pattern with peribronchial cuffing.

Affected ferrets received broad spectrum antimicrobial drugs, bronchodilators, expectorants, nonsteroidal anti-inflammatory drugs, and nebulization; all clinical signs except the dry cough temporarily decreased. Numerous ferrets from the distribution center were later surrendered to a ferret rescue and shelter operation, where their cough continued for as long as 4 years.

## Materials and Methods

### Affected Ferrets

In April 2009, a 2-year-old, spayed female ferret at the ferret rescue and shelter, which had originated from the breeding facility in Canada and passed through the US distribution center, became acutely dyspneic and died within 15 minutes. The ferret had shown signs of respiratory disease since arrival at the shelter. Previously at the shelter, 2 ferrets, 4.5 years of age, had shown chronic cough; 1 died of dyspnea in October 2010, and the other was euthanized for humane purposes in November 2010. Both had originated from the breeding facility and passed through the distribution center. Postmortem examinations were performed on all 3 ferrets. After the 2-year-old ferret died in April 2009, BAL samples and ocular swabs were obtained in July 2009 from 3 other ferrets with a history of respiratory disease since their arrival at the ferret shelter. For further diagnostic investigation, BAL samples and ocular swabs were collected from 9 additional affected ferrets in January 2010; one of these was the ferret that died in October 2010.

### Survey of Healthy Ferrets

At a large commercial breeding facility in which signs of respiratory disease had not been observed, BAL samples were obtained from 10 euthanized healthy male ferrets, 5 weeks to 5 years of age. Before postmortem examination, samples were collected from the euthanized ferrets by BAL through an incision in the caudal trachea. Nonbacteriostatic saline (10 mL/kg) was flushed into the caudal trachea and lungs and then recovered by aspiration into the syringe. That process was repeated 2× and the final flush fluid was submitted for bacterial culture. Complete postmortem examinations were performed, and sections of lung were collected for bacterial and mycoplasma culture. Additional tissue samples were collected from the lungs, trachea, nasal turbinates, brain, liver, kidneys, spleen, stomach, small and large intestine, thoracic and mesenteric lymph nodes, pancreas, and adrenal glands for routine histopathologic examination.

### Histologic and Immunohistochemical Analyses and Confocal Microscopy

From the 3 ferrets that died April 2009–November 2010, postmortem tissue samples (lungs, trachea, nasal turbinates, brain, liver, kidneys, spleen, stomach, small and large intestine, thoracic and mesenteric lymph nodes, pancreas, and adrenal glands) were collected. They were fixed in neutral-buffered, 10% formalin solution and processed by standard methods for histopathologic examination.

For immunohistochemical examination, paraffin-embedded samples of lung from the 3 ferrets that died were cut into 5-μm sections. An Enhanced Alkaline Phosphatase Red Detection Kit (Ventana Medical Systems, Inc., Tucson, AZ, USA) and bulk buffers specifically designed for use on the BenchMark Automated Staining System (Ventana Medical Systems, Inc.) were used for immunolabeling. Slides were baked in a drying oven at 60°C for 20 min, barcode labeled, and placed in the BenchMark for deparaffinization and heat-induced epitope retrieval. Slides were then incubated with a mouse monoclonal antibody against mycoplasma (primary antibody) (Chemicon, Billerica, MA, USA) at a concentration of 1:100 for 30 min. The monoclonal antibody was raised against *M. bovis* strain M23, but it is known to cross-react with numerous other mycoplasma species.

The slides were counterstained by using hematoxylin (Ventana Medical Systems, Inc.), then dehydrated, cleared, and mounted. For a positive control, we used formalin-fixed, paraffin-embedded sections of lung from an *M. bovis*–positive cow (tested by bacterial culture). For negative controls, we replaced the primary antibody with homologous nonimmune serum.

A Zeiss 510 microscope (Jena, Germany) was used for confocal imaging to acquire fluorescent images, and the Zeiss LSM image analysis software was used for characterizations. The images represented a differential interference contrast/Nomarski image with green (488 nm, argon laser excitation, fluorescein isothiocyanate [FITC]) and red (543 nm, rhodamine, helium–neon excitation, tetramethylrhodamine-5- [and 6-] isothiocyanate [TRITC])–labeled overlay to demonstrate localization of labels, as described, with slight modified according to Ubels et al. (*12*).

### Transmission and Scanning Electron Microscopy

For transmission electron microscopy, lung tissue samples that had been fixed in neutral-buffered, 10% formalin solution were trimmed into 2-mm pieces and postfixed in 1% osmium tetroxide in 0.1 M sodium phosphate buffer for 2 h. Tissues were serially dehydrated in acetone and embedded in Poly/Bed 812 resin (Polysciences Inc., Warrington, PA, USA) in flat molds. Sections were obtained with a Power Tome XL ultramicrotome (Boeckeler Instruments, Tucson, AZ, USA). To identify areas of interest, we stained semithin (0.5-μm) sections with epoxy tissue stain and examined them under a light microscope. Then we cut ultrathin (70-nm) sections, mounted them onto 200-mesh copper grids, stained them with uranyl acetate and lead citrate, and examined them under a 100 CXII transmission electron microscope (JEOL, Peabody, MA, USA).

For scanning electron microscopy, formalin-fixed lung tissues were trimmed into 2–4-mm pieces, postfixed for 1 h in 1% osmium tetroxide, and rinsed for 30 min in 0.1 M sodium phosphate buffer. Tissues were serially dehydrated in ethanol and dried in a critical point dryer (Model 010; Balzers, Witten, Germany) with liquid carbon dioxide as the transitional fluid. Samples were mounted on aluminum studs by using carbon suspension cement (SPI Supplies, West Chester, PA, USA). Samples were then coated with an osmium coater (NEOC-AT; Meiwa Shoji Co., Tokyo, Japan) and examined in a JSM-7500F (cold field emission electron emitter) scanning electron microscope (JEOL).

### Bacterial Cultures

We submitted 12 BAL samples and 12 ocular swab samples for bacterial and mycoplasma culture by standard microbiologic techniques. The samples were from live ferrets that originated from the distribution center and showed clinical signs of respiratory disease, including coughing. We also submitted 10 BAL samples from 10 healthy ferrets from a different commercial breeding facility not affected by respiratory disease.

### PCR and Sequence Analysis

Only mycoplasmas obtained from BAL samples were analyzed by PCR and nucleic acid sequencing. A plug of agar containing *Mycoplasma* spp. colonies was gouged from the surface of a mycoplasma agar plate by using a 10-μL disposable inoculation loop and transferred to a microcentrifuge tube. The agar plug was digested by addition of 200 μL of Buffer ATL (QIAGEN, Valencia, CA, USA) and 20 μL of proteinase K solution (QIAGEN), followed by overnight incubation at 56°C. DNA was extracted from the digest by using a DNeasy Blood and Tissue kit (QIAGEN) according to manufacturer’s instructions.

For PCR, we used 2 sets of primers selective for the bacterial 16S rDNA or the mycoplasma RNA polymerase B (*rpoB*) gene. The nucleic acid sequences for the 16s rDNA gene were 5′-AGAGTTTGATCMTGGCTCAG-3′ for the forward primer and 5′-GGGTTGCGCTCGTTR-3′ for the reverse primer; this primer set produced an amplicon of ≈1,058 bp. The nucleic acid sequences for the mycoplasma *rpoB* gene were 5′- GGAAGAATTTGTCCWATTGAAAC-3′ for the forward primer and 5′- GAATAAGGMCCAACACTACG-3′ for the reverse primer; this primer set produced an amplicon of ≈1,613 bp. The PCRs were performed by using Platinum Taq DNA Polymerase High Fidelity (Invitrogen Corp., Carlsbad, CA, USA). The reaction mixture consisted of 3 μL DNA; 1 unit of Platinum Taq DNA Polymerase High Fidelity; 60 mmol/L Tris-SO4 (pH 8.9); 18 mmol/L ammonium sulfate; 2 mmol/L magnesium sulfate; 0.2 mmol/L each of dATP, dCTP, dGTP and dTTP; 16.9 μL molecular biology grade water; and 0.5 µmol/L each of the PCR primer. The reaction conditions for the 16s rDNA gene were 1 cycle at 94°C for 4 min; 35 cycles at 94°C for 30 s, 58°C for 45 s, 68°C for 75 s; followed by a final extension step at 68°C for 5 min. The reaction conditions for the *rpoB* gene were 1 cycle at 94°C for 4 min; 40 cycles at 94°C for 45 s, 55°C for 45 s, 68°C for 90 s; followed by a final extension step at 68°C for 5 min.

The PCR products were stained with ethidium bromide and examined after electrophoresis through a 1.5% agarose gel. The PCR amplicons were excised from gels, purified by using the QIAquick Gel Extraction Kit (QIAGEN), and submitted to the Research Technology Support Facility at Michigan State University for nucleic acid sequencing. Several internal primers were designed to derive the complete sequences of the PCR amplicons. The derived sequences were edited by using Sequencher software (Gene Codes Corporation, Ann Arbor, MI, USA) and analyzed by using BLAST (www.ncbi.nlm.nih.gov/blast/Blast.cgi).

The nucleic acid sequences of the mycoplasma isolates and sequences from other *Mycoplasma* spp. obtained from GenBank were imported into the MEGA4 program (www.megasoftware.net), aligned by using ClustalW in the MEGA4 program, and subjected to phylogenetic analyses. For each isolate analyzed, 933 bp of the 16S rDNA gene sequence and 733 bp of the *rpoB* gene sequence were available. Phylogenetic trees were constructed by using the neighbor-joining method; data were resampled 1,000× to generate bootstrap percentage values.

## Results

### Gross and Histologic Lesions

**Figure 2:**
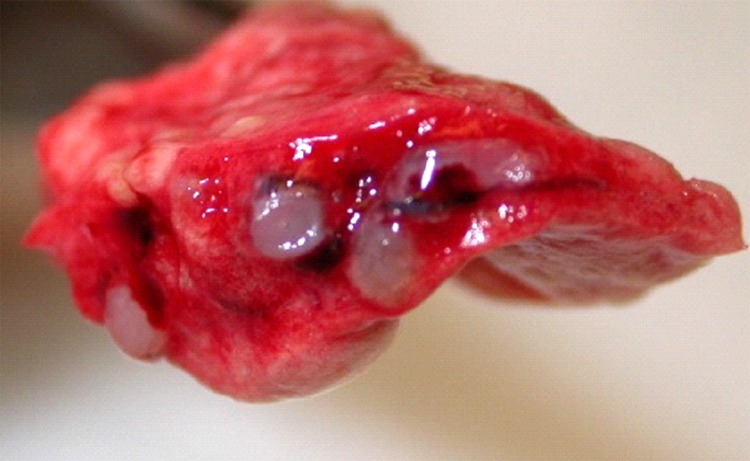
Lungs from a 2-year-old ferret that died of acute dyspnea, showing multifocal, tan to gray semifirm nodules centered on airways and severely narrowed lumina of affected airways.

**Figure 3:**
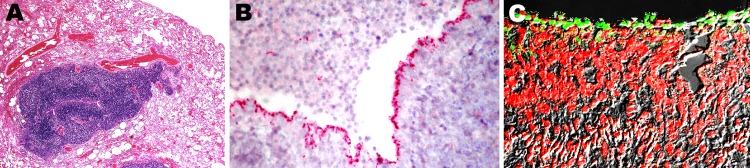
Micrographs of a section of lung from a 2-year-old ferret that died of acute dyspnea. A) Image shows moderate bronchointerstitial pneumonia with severe hyperplasia of bronchiole-associated lymphoid tissue around a narrowed airway lumen; original magnification ×40. B) Immunohistochemical analysis conducted with antibodies against mycoplasmas demonstrates intense labeling along the apical border of the ciliated respiratory epithelium; original magnification ×40. C) Confocal scanning laser microscopy conducted with antibodies against mycoplasmas demonstrates intense fluorescent labeling along the brush border of the bronchial epithelial cells; original magnification ×40.

Gross and histologic lesions from the 3 ferrets that died or were euthanized because of respiratory disease were similar and restricted to the lungs. The lungs were characterized by multifocal, tan to gray, somewhat firm nodules centered on airways randomly distributed throughout the pulmonary parenchyma (Figure 2). Hematoxylin and eosin–stained lung sections revealed a moderate bronchointerstitial pneumonia with severe bronchiole-associated lymphoid tissue (BALT) hyperplasia (Figure 3, panel A). BALT hyperplasia was commonly associated with marked narrowing of airway lumina. Additional findings included moderate perivascular lymphoid cuffing and diffuse pulmonary congestion. The lumina of some bronchi contained large amounts of mucus admixed with few sloughed epithelial cells and lymphocytes (catarrhal bronchitis).

Immunohistochemical examination (with antibodies against mycoplasmas) of affected lung tissue from all 3 ferrets that died exhibited strong labeling along the brush border of terminal respiratory epithelial cells (Figure 3, panel B). There was no penetration of organisms into the adjacent pulmonary parenchyma. With the same antibodies against mycoplasmas labeled with a fluorescent chromogen, confocal laser microscopy showed positive labeling along the apical border of the lining epithelium of terminal airways (Figure 3, panel C). Additional immunohistochemical examination and reverse transcription PCR for canine distemper and influenza A viruses, performed on samples of lung from all 3 ferrets that died, detected no virus.

**Figure 4:**
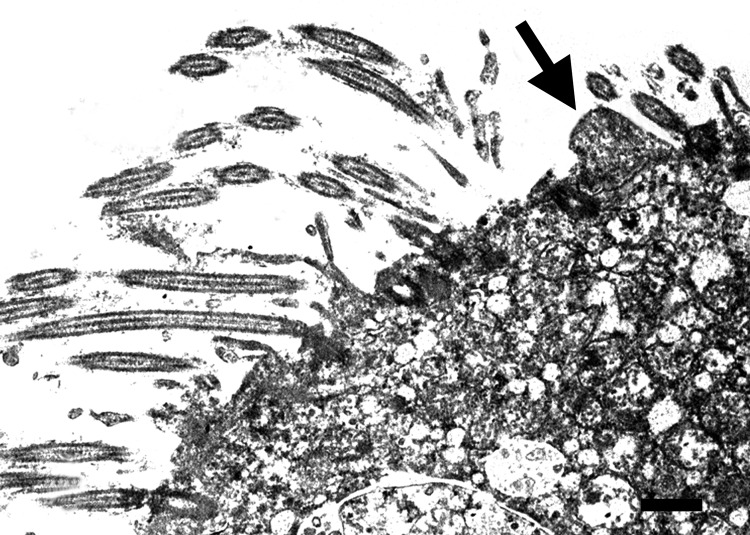
Transmission electron micrograph of the lung from a 2-year-old ferret that died of acute dyspnea, showing loss of cilia in bronchial epithelial and cellular degeneration characterized by swelling of endoplasmatic reticulum, vacuolization of mitochondria with loss of christae, and intranuclear chromatin dispersion. Attached to the apical surface of a ciliated cell is a 0.8-μm pleomorphic mycoplasma-like organism (arrow). Scale bar = 0.5 µm.

Transmission electron microscopy showed bronchial epithelial cells with loss of cilia and cellular degeneration characterized by swelling of endoplasmic reticulum, vacuolization of mitochondria with loss of christae, and intranuclear chromatin dispersement. Attached to the apical surface of a ciliated cell were pleomorphic, round to ovoid, ≈0.8-µm mycoplasma-like organisms (Figure 4).

**Figure 5:**
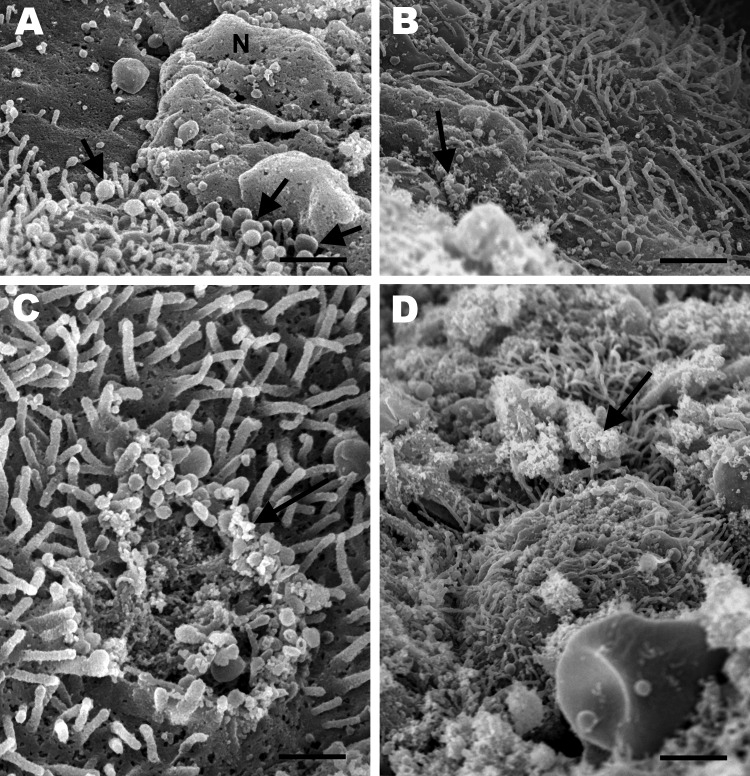
Scanning electron micrographs of the lung from a 2-year-old ferret that died of acute dyspnea, showing A) marked loss of cilia with multifocal degenerative changes characterized by bulbous swelling of cilia (arrows) and necrosis of bronchial epithelial cells (N) (scale bar = 1 µm); B) marked loss of cilia and numerous pleomorphic mycoplasma-like organisms diffusely attached to the mucosal surface (arrow) (scale bar = 1.25 µm); C) focal area of cilia loss and cell membrane damage with mycoplasma-like organisms (arrow) at the periphery of the lesion (scale bar = 400 nm); and D) many mycoplasma-like organisms (arrow) covering ciliated bronchial epithelial cells (scale bar = 2 µm).

Electron microscopy showed severe denudation of bronchial epithelial cells. Cilia were commonly lost or had undergone degenerative changes characterized by bulbous swelling (Figure 5, panel A). Many necrotic bronchial epithelial cells were adhered to the luminal surface, and many pleomorphic mycoplasma-like organisms were diffusely attached to the mucosal surface of bronchi and bronchioles (Figure 5, panel B). In some areas, focal loss of cilia and cell membrane damage and mycoplasma-like organisms were observed along the periphery of such lesions (Figure 5, panel C). In other areas, the mucosal surface was covered by many mycoplasma-like organisms that completely obscured the cilia (Figure 5, panel D). Among the 10 healthy ferrets, no gross or histologic lesions suggestive of mycoplasma infection were identified.

### Bacteria

The 12 BAL samples from affected ferrets were all positive for fast-growing, glucose-fermenting mycoplasmas but negative for other bacteria. Ocular swabs from these ferrets were negative for bacteria. No bacteria or mycoplasmas were isolated from the 10 healthy ferrets.

### PCR and Sequences

**Figure 6:**
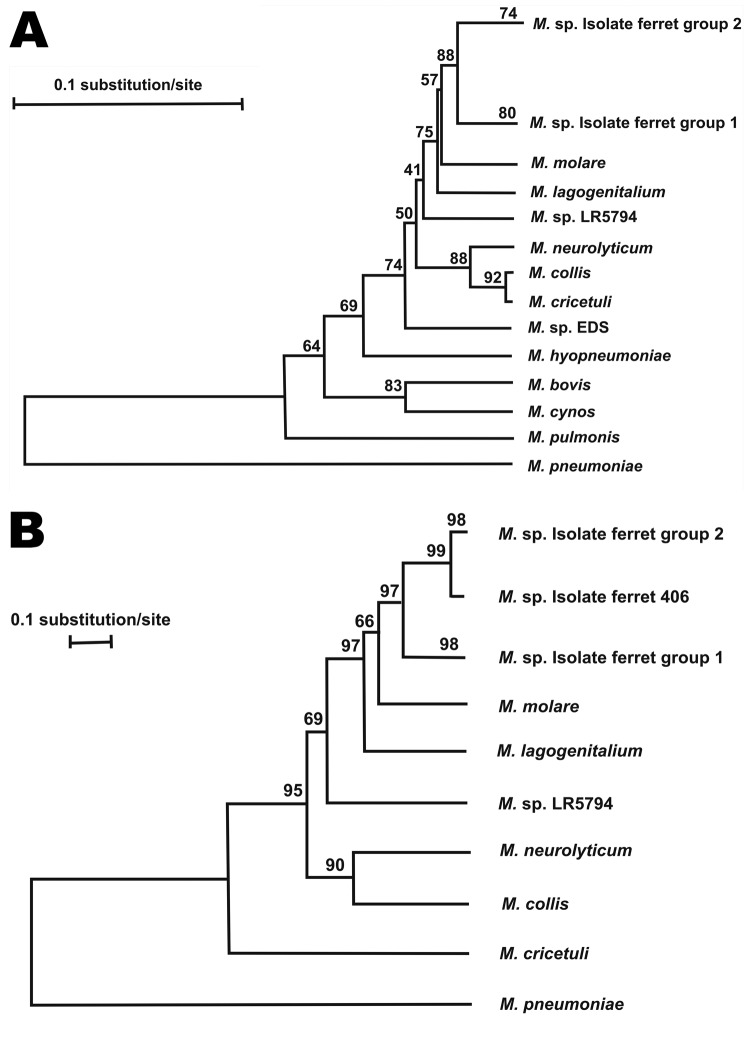
Phylogenetic analysis of A) partial 16S rDNA gene (933 bp) and B) partial RNA polymerase B gene (733 bp) for the new mycoplasma isolates and other closely related *Mycoplasma *species as conducted in MEGA4 (*13*). The bootstrap consensus phylogenetic trees were constructed by using the neighbor-joining method (*14*). The bootstrap values as shown above the branches were inferred from 1,000 replicates of data resampling to represent the evolutionary distances of the species analyzed (*15*). The tree is drawn to scale; branch lengths are in the same units as those of the evolutionary distances used to infer the phylogenetic tree (i.e., the units of the number of base substitutions per site).

Analyses of nucleic acid sequences from the 16S rDNA gene (GenBank accession nos. JQ910955– JQ910966) for each of the 12 mycoplasma isolates showed that the isolates were 99% similar to each other and segregated the isolates into 2 groups defined by nucleotide differences at 3 positions. Phylogenetic analysis with partial 16S rDNA gene sequences showed that the isolates were 96% to 97% similar to *M. molare* (isolated from a canid). Other closely related *Mycoplasma* spp. included *M. lagogenitalium* (isolated from Afghan pika), *M. neurolyticum* (isolated from mice and rats), *M.* sp. LR5794 (isolated from raccoons), *M. collis* (isolated from mice and rats), *M. cricetuli* (isolated from Chinese hamsters), and *M.* sp. EDS (isolated from house musk shrews) (Figure 6, panel A). On the basis of the 16S rDNA gene sequences, these mycoplasmas isolated from ferrets, along with the aforementioned closely related *Mycoplasma* spp., are in the hominis group of mycoplasmas.

Analyses of nucleic acid sequences from the *rpoB* gene (GenBank accession nos.. JQ91067–JQ91078) for each of the mycoplasma isolates from ferrets segregated the isolates into 2 groups of genetic variants (groups 1 and 2), which were 90% –91% similar to each other. Within a group, the isolates were 99%–100% or 98%–100% similar to each other. Although nucleotide differences were identified in as many as 12 positions within a group and 65 positions between groups, the corresponding amino acid sequences were 100% similar within a group and differed at only 2 aa positions between groups. Phylogenetic analysis showed that the partial *rpoB* gene sequences of the isolates were only 85%–86% similar with *M. molare* and 84%–86% similar to *M. lagogenitalium,* the most closely related *Mycoplasma* species. (Figure 6, panel B). Grouping of the isolates according to sequences of 16S rDNA and *rpoB* gene were in agreement for all but 1 isolate. Phylogenetic relatedness of these newly identified mycoplasmas to other *Mycoplasma* spp. was similar for the 16s rDNA and the *rpoB* genes.

## Discussion

*Mycoplasma* spp. are the smallest free-living prokaryotic microorganisms of the class Mollicutes (*16*). They lack a cell wall and are thought to have developed from genome reduction of gram-positive bacteria (*17*). Most species are host-specific facultative anaerobes and do not usually replicate in the environment (*18*). Their complex growth requirements include cholesterol, fatty acids, and amino acids (*19*). In the respiratory tract, mycoplasmas attach to ciliated epithelial cells by surface-exposed adhesions (*20*). Although the pathogenesis of host cell injury remains largely unknown, proposed virulence mechanisms include induction of proinflammatory cytokines by phagocytes (*21*), oxidative damage to host cells by production of toxic by-products (*22*), and cleavage of host DNA through nucleases (*23*). Many mycoplasmas cause B lymphocytes and/or T lymphocytes to commence dividing in a nonspecific manner (*24*). This mitogenic effect probably explains the characteristic BALT hyperplasia observed in infected host tissues. A commonly described strategy for immune evasion is phenotype plasticity, whereby reversible switching or modification of membrane protein antigens results in altered surface antigens (*25*). This mechanism might support the persistent, chronic nature of mycoplasmosis often observed. The precise role of mycoplasmas in various host species is often difficult to interpret because certain mycoplasmas can be isolated from apparently healthy animals.

The data presented here describe a recently emerging respiratory disease of ferrets, characterized especially by high morbidity rates and a dry, nonproductive cough, associated with an infection by a novel *Mycoplasma* species. To our knowledge, no *Mycoplasma* species have been associated with clinical disease in ferrets or other mustelids. On the basis of limited sequence data, the isolated mycoplasmas most likely represent a novel *Mycoplasma* specie or species.

In 1982, a study from Japan reported isolation of a glucose-fermenting mycoplasma from the oral cavities of 81% of clinically healthy ferrets kept in a laboratory setting (*26*). This mycoplasma isolate was not antigenically related to any reference strains from dogs, cats, sheep, cattle, mice, raccoon dogs, or a Japanese badger. In 1983, similarly fast-growing, glucose-fermenting mycoplasmas were isolated from the lungs of healthy mink kits (1–2 months of age) in Denmark (*27*). This species was named *M. mustelae*. Because the *Mycoplasma* spp. isolates from healthy ferrets or mink were not genetically characterized, comparison with the isolates from ferrets with respiratory disease in this study was not possible. The *Mycoplasma* species isolated from affected ferrets showed the highest sequence similarity to *M. molare* and *M. lagogenitalium*. *M. molare* was first isolated in 1974 from the pharynx of dogs with mild respiratory disease (*28*). However, the pathogenicity of *M. molare* in dogs or other species remains speculative. *M. lagogenitalium* was first isolated in 1997 from prepucial samples from apparently healthy Afghan pikas (*29*).

The 3 mycoplasma isolates obtained from BAL samples from 3 ferrets in July 2009 were highly homogenous according to limited sequence data. All 3 isolates were included in the mycoplasma isolate group 2. Of note, only 3 of the 9 isolates obtained from BAL samples from 9 ferrets in January 2010 had the same partial *rpoB* amino acid sequence data as the previous isolates and were also included in the mycoplasma isolate group 2. In contrast, the partial *rpoB* sequence of 5 of the more recent isolates differed by 9%–10% from that of the previous isolates, and the isolates were identified as belonging to mycoplasma isolate group 1. Whether these differences represent multiple *Mycoplasma* species circulating through the ferret population or a genetic change of the original mycoplasma over time is uncertain, as is the virulence of each of the potential strains. Only 1 isolate was identified in the bronchoalveolar lavage sample from a ferret for which postmortem examination confirmed lesions consistent with a mycoplasma infection. Experimental reproduction with the different isolates is required to further elucidate the virulence of each putative novel mycoplasma.

Respiratory disease attributed to mycoplasma infections in cattle (*30*), pigs (*31*), poultry (*32*), mice, and rats has been well described (*33*). The clinical signs and microscopic lesions in ferrets with the emerging respiratory disease described here closely resembled signs and lesions described for pigs infected with *M. hyopneumoniae* (*34*), rats infected with *M. pulmonis* (*35*), and cattle infected with *M. bovis* (*36*). For all of these species, chronic pulmonary mycoplasmosis is characterized by lymphoplasmacytic perivascular cuffing and extensive BALT hyperplasia, as was observed in ferrets in this study. Furthermore, *M. cynos* (*37*) and an untyped *Mycoplasma* species (*38*) reportedly cause pulmonary lesions similar to those in dogs and cats, respectively.

The similarity between the pathologic changes in the ferrets and those in other species with mycoplasmal pneumonia highly supports a causal relationship between the pulmonary disease and the identified novel mycoplasma in these ferrets. In addition, mycoplasmas were the only bacterial pathogens recovered from the respiratory tract of diseased ferrets, there was no microscopic evidence of a viral disease, and immunohistochemical and reverse transcription PCR results for canine distemper and influenza A were negative. Furthermore, mycoplasmas were not detected in the sampled population of healthy domestic ferrets 5 weeks to 5 years of age.

Because mycoplasmas have been recovered from the respiratory tract of apparently healthy mustelids (*26*,*27*), other unknown factors might have predisposed the lungs of these ferrets to colonization. The severity of the clinical signs might have been exacerbated by infections with secondary bacteria, as commonly occurs in other species (*30*,*31*,*33*), and antimicrobial drug therapy might have prevented isolation of such bacteria. A concurrent viral disease seems unlikely because characteristic microscopic lesions were absent and common respiratory viral pathogens in ferrets were not identified. We speculate that the stress of shipment from the breeding facility to the distribution center might have resulted in the disease manifestation. To more fully elucidate pathogenicity and disease dynamics in this species, experimental reproduction of the respiratory disease in ferrets is necessary.
